# Supplementary material for: Mapping-by-Sequencing via MutMap Identifies a Mutation in ZmCLE7 Underlying Fasciation in a Newly Developed EMS Mutant Population in an Elite Tropical Maize Inbred
Source: Genes (Basel). 2020 Mar 6;11(3):281. doi: 10.3390/genes11030281 (PMC7140824; doi:10.3390/genes11030281)
Supplement: Supplementary file 1 [file genes-11-00281-s001.zip › supplementary ZmCLE7 ok/Figure S1 SV.docx]

**Figure S1. Nucleotide alignment of the CLE7 locus among reference B73, unmutagenized ML10 and E1-9 mutant.**

All highlights were made on B73 sequence. The difference between B73 and ML10, which were shared by E1-9, were highlighted in yellow. ATG start codon highlighted in red, intron highlighted in green, TAG stop codon highligted in pink. The promoter deletion in E1-9 was highlighted in Turquoise

MUSCLE (3.8) multiple sequence alignment

B73 GAGTTTCAGAACTAAATGATACCCGTATAGACAAGTTTGGCAACCTGAGTATACATATTT

ML10 GAGTTTCAGAACTAAATGATACCCGTATAGACAAGTTTGGCAACCTGAGTATACATATTT

E1-9 GAGTTTCAGAACTAAATGATACCCGTATAGACAAGTTTGGCAACCTGAGTATACATATTT

************************************************************

B73 TAGGCCTAGAGATTCGTGTGGCACCATAAAGTTAG-TTTTTTTTTAAAAAAAACCAATCA

ML10 TAGGCCTAGAGATTCGTGTGGCACCATAAAGTTAGTTTTTTTTTTAAAAAAAACCAATCA

E1-9 TAGGCCTAGAGATTCGTGTGGCACCATAAAGTTAGTTTTTTTTTTAAAAAAAACCAATCA

*********************************** ************************

B73 TGCACTTTAACTATTACATTCACTCTTTTGGCTTTCATAAAACTTTGTGTGCTTAGCAGC

ML10 TGCACTTTAACTATTACATTCACTCTTTTCGCTTTCATAAAACTTTGTGTGCTTAGCAGC

E1-9 TGCACTTTAACTATTACATTCACTCTTTTCGCTTTCATAAAACTTTGTGTGCTTAGCAGC

***************************** ******************************

B73 ATGCAGCAGCACGTGTGGCATATATGAAAT----TATAATGCAA----AGCTAGCTAGTA

ML10 ATGCAGCAGCACGTGTGGCATATATGAAATTATATATAATGCAAAGCTAGCTAGCTAGTA

E1-9 ATGCAGCAGCACGTGTGGCATATATGAAATTATATATAATGCAAAGCTAGCTAGCTAGTA

****************************** ********** ************

B73 GATTCTACTACTGTCGTACATTCTCATCCGGCGCTAAAACGCTACACAAGTGGGCTGTTG

ML10 GATTCTACTACTGTCGTACATTCTCATCCGGCGCTAAAACGCTACACAAGTGAGCTGTTG

E1-9 GATTCTACTACTGTCGTACATTCTCATCCGGCGCTAAAACGCTACACAAGTGAGCTGTTG

**************************************************** *******

=B73 TATGTACTTGTACTTTACTCCGTAGAGGAAGGGGATAAAGATTGTATCGTCGTACTAGCT

ML10 TATGTACTTGTACTTTACTCCGTAGAGGAAGGGGATAAAGATTGTATCGTCGTACTAGCT

E1-9 TATGTACTTGTACTTTACTCCGTAGAGGAAGGGGATAAAGATTGTATCGTCGTACTAGCT

************************************************************

B73 CTAGTATGTATGTGGCAGCGCGCGGTACCTTTGATTCTTTCTTTTTGTCTTCACATGACT

ML10 CTAGTATGTATGTGGCAGCGCGCGGTACCTTTGATTCTTTATTTTTGTCTGCACATGACT

E1-9 CTAGTATGTATGTGGCAGCGCGCGGTACCTTTGATTCTTTATTTTTGTCTGCACATGACT

**************************************** ********* *********

B73 AGCAGTAGTAGCCAGCTTGTTGAACTTGACGTTGTTCAACCATGGCTGTGATGTGACCCT

ML10 AGCAGTAGTAGCCAGCTTGTTGAACTTGACGTTGTTCAACCATGGCTGTGATGTGACCCT

E1-9 AGCAGTAGTAGCCAGCTTGTTGAACTTGACGTTGTTCAACCATGGCTGTGATGTGACCCT

************************************************************

B73 CACAAACACGTCAGAGCAAGCAAAAGATGCGGTCATCAACTCGCTTTCCAAACTGATGCG

ML10 CACAAACACGTCAGAGCAAGCAAAAGATGCGGTCATCAACTCGCTTTCCAAACTGATGCG

E1-9 CACAAACACGTCAGAGCAAGCAAAAGATGCGGTCATCAACTCGCTTTCCAAACTGATGCG

************************************************************

B73 TACTGGATCCGCGCGCAGGGCCAGGATCTCCACTCCACTCCATTGCAGGTGCCAGTCTTT

ML10 TACTGGATCCGCGCGCAGGGCCAGGATCTCCACTCCACTCCATTGCAGGTGCCAGTCTTT

E1-9 TACTGGATCCGCGCGCAGGGCCAGGATCTCCACTCCACTCCATTGCAGGTGCCAGT----

********************************************************

B73 ACTTCACATGATCCTCATGGAAACAGATCCTCTGCGGTACGTCTTCTGTGGATGTAGCCC

ML10 ACTTCACATGATCCTCATGGAAACAGATCCTCTGCGGTACG---TCTGTGGATGTAGCCC

E1-9 ---------------------------TCCTCTG--------------------------

*******

B73 ACGCCGGAGTCACTGCCCGCGTGCCCGTCTCCGGCCCCACGCGCAGCGACTACGTCCGCC

ML10 ACGCCGGAGTCACTGCCCGCGTGCCCGTCTCCGGCCCCACGCGCAGCGACTACGTCCGCC

E1-9 ------------------------------------------------------------

B73 TAAGAAGAGGGAGAGATACATGAATACCGTATGTGTTTTTGTTATTAGAGAAGCTGGAGG

ML10 TAAGAAGAGGGAGAGATACATGAATACCGTATGTGTTTTTGTTATTAGAGAAGCTGGAGG

E1-9 ------------------------------------------------------------

B73 CGGTGGGTCTCCTATAAGTACCGCCGGGGCCCCCCCTCCTCATATATATGTTGGTCGTCG

ML10 CGGTGGGTCTCCTATAAGTACCGCCGTGGCCCCCCCCTCCT---CATATGTTGGTCGTCG

E1-9 ------------------------------------------------------------

B73 TCATCCTAGAAGCGCAAGCCTAGCTGGTGCACTCACGTCTCTCCACACACCACTGCTAAG

ML10 TCATCCTAGAAGCGCAAGCCTAGCTGGTGCACTCACGTCTCTCCACACACCACTGCTAAG

E1-9 ------------------------------------------------------------

B73 ACTGCCGGCGAGTCCTCCGGCCGGTTATACACACCGCGGTTTTTGGTCGTCGTCACAAAC

ML10 ACTGCCGGCGAGTCCTCCGGCCGGTTATACACACCGCGGTTTTTGGTCGTCGTCACAAAC

E1-9 ------------------------------------------------------------

ATG start codon

B73 CCAACCGTCAGCCATGGCCCGGATCTTCCTCTGCATGAGCTTGGCGGCGGCGTGCTGCTG

ML10 CCAACCGTCAGCCATGGCCCGGATCTTCCTCTGCATGAGCTTGGCGGCGGCGTGCTGCTG

E1-9 ------------CATGGCCCGGATCTTCCTCTGCATGAGCTTGGCGGCGGCGTGCTGCTG

************************************************

Intron 1

B73 CTTCTCCATTGCGCTGCTTCCACCGCCGGCGCAGGGCCGTCCTGGTGGGTGGTACGCCGC

ML10 CTTCTCCATTGCGCTGCTTCCACCGCCGGCGCAGGGCCGTCCTGGTGGGTGGTACGCCGC

E1-9 CTTCTCCATTGCGCTGCTTCCACCGCCGGCGCAGGGCCGTCCTGGTGGGTGGTACGCCGC

************************************************************

B73 AGCTTTTTTTTTCCCTATGCCGAACTTTATTAGTTTTCGTATGTCTGGGAACTGCTCCCT

ML10 AGCTTTTTTTTTCCCTATGCCGAACTTTATTAGTTTTCGTATGTCTGGGAACTGCTCCCT

E1-9 AGCTTTTTTTTTCCCTATGCCGAACTTTATTAGTTTTCGTATGTCTGGGAACTGCTCCCT

************************************************************

B73 GTCCTCCATTATATCCCCTCTCGCTTTCCTTTGACATTATTCTGGTGGTGTAGCAACTAG

ML10 GTCCTCCATTATATCCCCTCTCGCTTTCCTTTGACATTATTCTGGTGGTGTAGCAACTAG

E1-9 GTCCTCCATTATATCCCCTCTCGCTTTCCTTTGACATTATTCTGGTGGTGTAGCAACTAG

************************************************************

B73 CACTTGATGAATGTGCTATTGCATGTTCTTGAATCCTTGATGAATGCATGCATGTACTGT

ML10 CACTTGATGAATGTGCTATTGCATGTTCTTGAATCCTTGATGAATGCATGCATGTACTGT

E1-9 CACTTGATGAATGTGCTATTGCATGTTCTTGAATCCTTGATGAATGCATGCATGTACTGT

************************************************************

B73 GGTCGAGTTCTTTCGTTCTCATCTCTTTTTTTCTTTCTTTGTGTGTGTTTGGTTTTTCAG

ML10 GGTCGAGTTCTTTCGTTCTCATCTCTTTTTTTCTTTCTTTGTGTGTGTTTGGTTTTTCAG

E1-9 GGTCGAGTTCTTTCGTTCTCATCTCTTTTTTTCTTTCTTTGTGTGTGTTTGGTTTTTCAG

************************************************************

B73 GGCTGCCAGCCGGCGGCCGCATAAATCATCTGCCAGAGCCAACCACAGAGGTACCGCACG

ML10 GGCTGCCAGCCGGCGGCCGCATAAATCATCTGCCAGAGCCAACCACAGAGGTACCGCACG

E1-9 GGCTGCCAGCCGGCGGCCGCATAAATCATCTGCCAGAGCCAACCACAGAGGTACCGCACG

************************************************************

Intron 2

B73 TTCCGTTTATTGTTTCCGTTTCTTTATCGGTATGTATATAAATAAAAAAACCCCTACATA

ML10 TTCCGTTTATTGTTTCCGTTTCTTTATCGGTATGTATATAAATAAAAAAAACCCTACATA

E1-9 TTCCGTTTATTGTTTCCGTTTCTTTATCGGTATGTATATAAATAAAAAAAACCCTACATA

************************************************** *********

B73 CTGCCATCTACTCAAGCACTCTAGCGTTTCACTATATACCTGTACTGACATCTGTGTGCT

ML10 CTGCCATCTACTCAAGCACTCTAGCGTTTCACTATATACCTGTACTGACATCTGTGTGCT

E1-9 CTGCCATCTACTCAAGCACTCTAGCGTTTCACTATATACCTGTACTGACATCTGTGTGCT

************************************************************

B73 GGTGCAATGGAACTGACTGTCAGCCGGCCGCGGCGGCGGAGCAGGAGCAGCAGCAGCGCG

ML10 GGTGCAATGGAACTGACTGTCAGCCGGCCGCGGCGGCGGAGCAGGAGCAGCAGCAGCGCG

E1-9 GGTGCAATGGAACTGACTGTCAGCCGGCCGCGGCGGCGGAGCAGGAGCAGCAGCAGCGCG

************************************************************

B73 GCGTGCAGGTGAGGAAGACGAGGCCGGCATGGTCGCCAGCGGCGGCGGCGGAGGGGAGCG

ML10 GCGTGCAGGTGAGGAAGACGAGGCCGGCATGGTCGCCAGCGGCGGCGGCGGAGGGGAGCG

E1-9 GCGTGCAGGTGAGGAAGACGAGGCCGGCATGGTCGCCAGCGGCGGCGGCGGAGGGGAGCG

************************************************************

B73 TGAGGCCGGAGATGCGGGCGGTGCCCGGGGGGCCAGACCCGCTGCACCACCACGGCGGCA

ML10 TGAGGCCGGAGATGCGGGCGGTGCCCGGGGGGCCAGACCCGCTGCACCACCACGGCGGCA

E1-9 TGAGGCCGGAGATGCGGGCGGTGCCCGGGGGGCCAGACCCGCTGCACCACCACGGCGGCA

************************************************************

B73 GCCCCAGCAGGCGGCCTGCAGCAGGGACGCACCCGGTGACCCGGCCGGCCGGCCAGCCGG

ML10 GCCCCAGCAGGCGGCCTGCAGCAGGGACGCACCCGGTGACCCGGCCGGCCGGCCAGCCGG

E1-9 GCCCCAGCAGGCGGCCTGCAGCAGGGACGCACCCGGTGACCCGGCCGGCCGGCCAGCCGG

************************************************************

TAG stop codon

B73 CATGGCTAGGCTAG

ML10 CATGGCTAGGCTAG

E1-9 CATGGCTAGGCTAG

**************
